# Rapid Viral Diagnosis of Orthopoxviruses by Electron Microscopy: Optional or a Must?

**DOI:** 10.3390/v10040142

**Published:** 2018-03-22

**Authors:** Hans R. Gelderblom, Dick Madeley

**Affiliations:** 1 (ret) Robert Koch Institute, Centre for Biological Threats and Special Pathogens, ZBS 4: Advanced Light and Electron Microscopy, Seestrasse 10, D-13353 Berlin, Germany; 2 (ret) University of Newcastle upon Tyne, Burnfoot, Stocksfield, Northumberland, NE43 7TN, UK; dickmadeley@aol.com

**Keywords:** diagnostic electron microscopy (DEM), rapid viral diagnosis, febrile vesicular rashes, skin lesions, orthopoxviruses (OPV), parapoxviruses (PPV), herpesviruses (HSV; VZV), negative staining

## Abstract

Diagnostic electron microscopy (DEM) was an essential component of viral diagnosis until the development of highly sensitive nucleic acid amplification techniques (NAT). The simple negative staining technique of DEM was applied widely to smallpox diagnosis until the world-wide eradication of the human-specific pathogen in 1980. Since then, the threat of smallpox re-emerging through laboratory escape, molecular manipulation, synthetic biology or bioterrorism has not totally disappeared and would be a major problem in an unvaccinated population. Other animal poxviruses may also emerge as human pathogens. With its rapid results (only a few minutes after arrival of the specimen), no requirement for specific reagents and its “open view”, DEM remains an important component of virus diagnosis, particularly because it can easily and reliably distinguish smallpox virus or any other member of the orthopoxvirus (OPV) genus from parapoxviruses (PPV) and the far more common and less serious herpesviruses (herpes simplex and varicella zoster). Preparation, enrichment, examination, internal standards and suitable organisations are discussed to make clear its continuing value as a diagnostic technique.

## 1. Introduction

There used to be a criticism of diagnostic virology that by the time the result was known the patient was either dead or better. That it could be of practical use is illustrated by the following episode. In the 1960s, before smallpox had been eradicated from the world, an adult male was found in the busy out-patient department (OPD) of a major London hospital late on a Friday where he had been all afternoon. He said he had just returned from East Africa where smallpox was still endemic and claimed that he had had chickenpox as a child and that he had not been vaccinated. The staff of the OPD were faced with a man with a widespread vesicular skin rash and a slight fever and a dilemma—was this a case of smallpox? Should they alert the media to warn everyone who had been in the OPD to contact their own doctor over the weekend, or was there a way to defuse the situation quickly? One of us (DM), as an electron microscopist at a different London hospital, was telephoned to ask if a quick diagnosis could be made. After advice about collecting specimens, a taxi arrived soon afterwards and a doctor from the other hospital got out, carrying a small syringe containing fluid aspirated from the patient’s vesicles as if it was an unexploded bomb.

In the electron microscopy (EM) laboratory, ten minutes later numerous herpesvirus-like particles were seen in the specimen and everyone could relax. Despite the patient’s claim to have had chickenpox in childhood, it was clear that chickenpox was what he now had. No national alert was necessary, with all the widespread anxiety that would have followed. The speed and certainty of the diagnosis by EM offered a lesson that still has relevance.

Four valuable lessons can be drawn from this episode: (1) That a useful diagnosis can be made in minutes from the arrival of the specimen; (2) Diagnostic EM (DEM) required no specific reagents (such as primers or antisera) or special equipment. All that is required was an electron microscope, microscope support grids, stain and a competent virologist familiar with the appearance of relevant viruses; (3) That seeing is believing—knowing what the causative virus looks like is a useful confirmation for any other tests added later; (4) That other, more serious, causes could be discounted.

With concerns over the possible re-emergence of poxvirus infections, either through the spread of existing animal viruses to susceptible humans [1,2,3,4,5], survival and escape of old material [6,7,8], molecular manipulation and synthetic biology [9] or even bioterrorism [10,11,12], there will be a need to provide accurate and quick diagnosis. This paper presents the possibilities and advantages of DEM and how DEM can help with organised future preparedness.

## 2. Orthopoxviruses (OPV), Herpesviruses and Other Agents

The major source of anxiety over unexpected skin infections of unknown aetiology will be smallpox because of its possible fatal outcome and potential to cause a serious epidemic and panic in the community. Smallpox, caused by the variola virus, has been eradicated as an endemic pathogen from the world’s population since 1980 [1] but stocks of the virus remain under WHO supervision under high-security at the CDC (Centers for Disease Control & Prevention), Atlanta, USA, and at VECTOR (State Research Center of Virology and Biotechnology), Koltsovo-Novosibirsk, Russia, for further research. Nevertheless, some variola virus may yet remain elsewhere, forgotten in a deep-freeze [9] or as dried crusts, and escape, or a virulent variant may be generated through laboratory molecular manipulation. Other viruses also cause vesicular lesions which may mimic smallpox. In addition, other micro-organisms (including several virus families) or some non-infective conditions may cause similar skin lesions as listed in Table 1 and Table 2.

The poxvirus family comprises two subfamilies: the *Chordopoxvirinae,* specific for the vertebrates, and the *Entomopoxvirinae,* specific for insects. Consequent to the progress in molecular techniques, chordopoxviruses are currently classified into 10 genera [13], with the genus orthopoxviruses (OPV) being most relevant for man and many higher animals (for reviews see: [1,4,14]).

**Figure 1:**
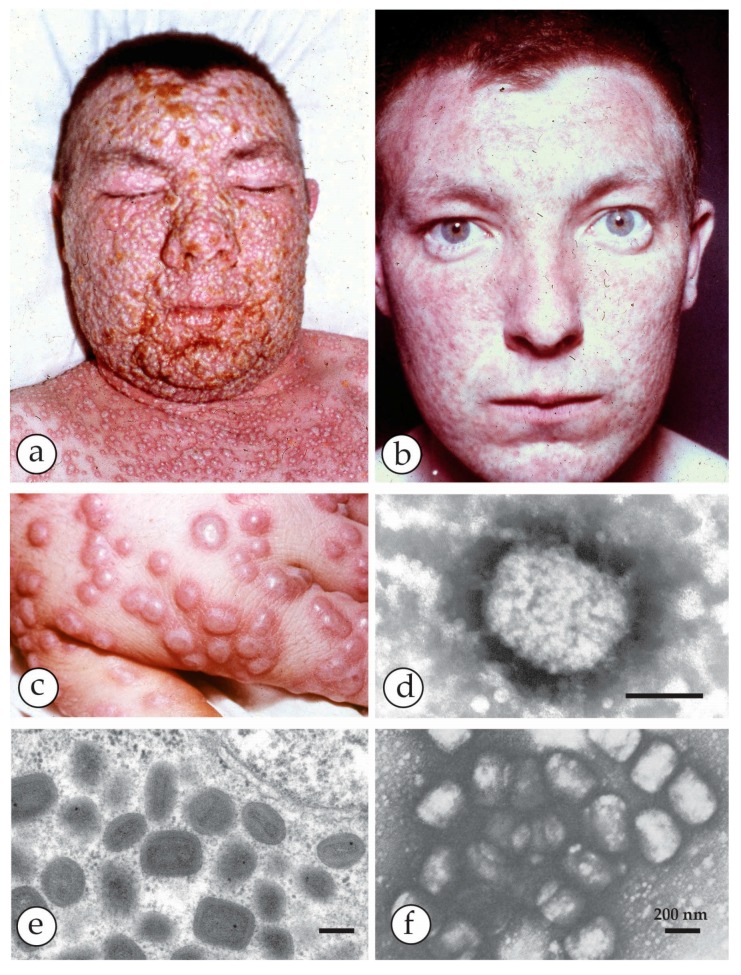
Last cases of endemic smallpox in Europe: (**a**,**b**) Smallpox patient from the 1962 outbreak in South Wales. (**a**) At the acute stage, with marked facial oedema, and (**b**) after recovery. He was 17 at the time, but looked middle-aged in the first photo. (**c**) The hand of one of the cases showing deep-seated, compact vesicles, often umbilicated and mostly at the same developmental stage. (**d**) Negative staining (NS) DEM of vesicle fluid with PTA (potassium phosphotungstic acid) revealed brick-shaped particles of the proper OPV size. (**e**,**f**) Thin section (TS) TEM (Transmission Electron Microscopy) and DEM of the last smallpox case in Germany [30]. (**e**) Virus was isolated on the chorio-allantoic membrane (CAM) and showed in ultrathin sections different cuts through fully assembled OPV. For more detail see Figure 14a,b. (**f**) direct DEM of vesicle fluid showed an abundance of typical OPV particles. (**f**) with kind permission of Thieme Verlag, Stuttgart, Germany. Bars (**d**–**f**) = 200 nm.

**Figure 2:**
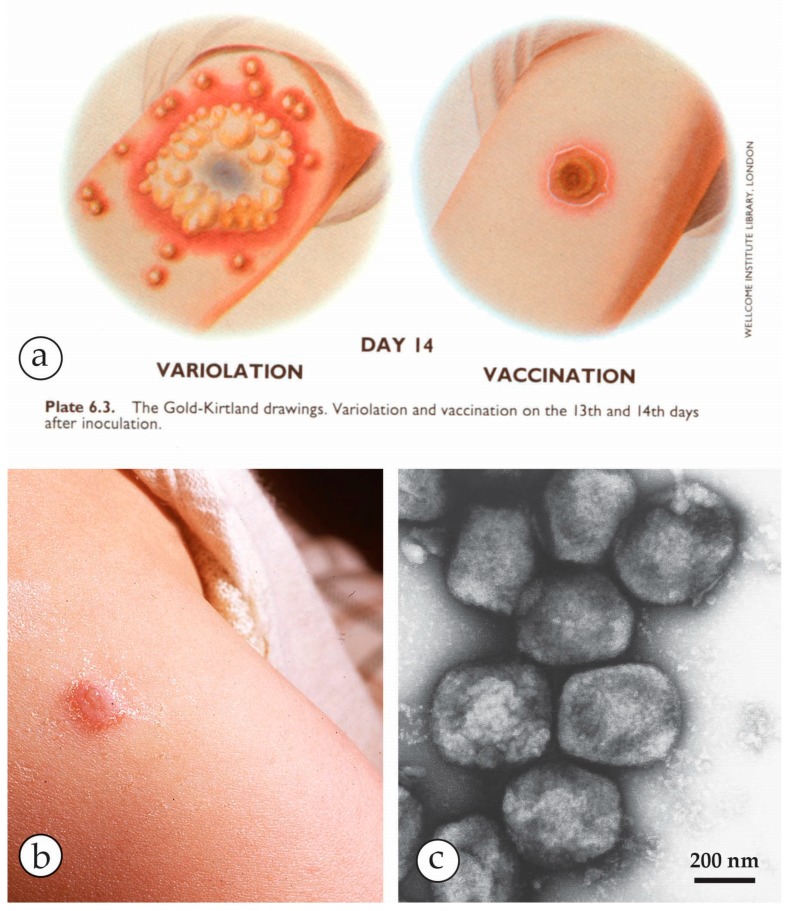
Variolation versus vaccination: (**a**) Variolation, the inoculation of small amounts of live variola lesion fluids was widely used as a protective, though highly risky measure, before Edward Jenner in 1796 established the much less risky vaccination using material from a cowpox lesion [31]. Plate from Fenner et al.: Smallpox and its Eradication [1] with kind permission of WHO, Geneva. (**b**) A routine primary vaccination lesion in a one-year old, containing little vesicle fluid. Photo taken after 1 week. (**c**) Vaccinia virus after NS showing all morphological criteria: size, shape and surface details of OPV. The origin of present day vaccines strains is heterogeneous. For details see [1,16,24,32].

**Figure 3:**
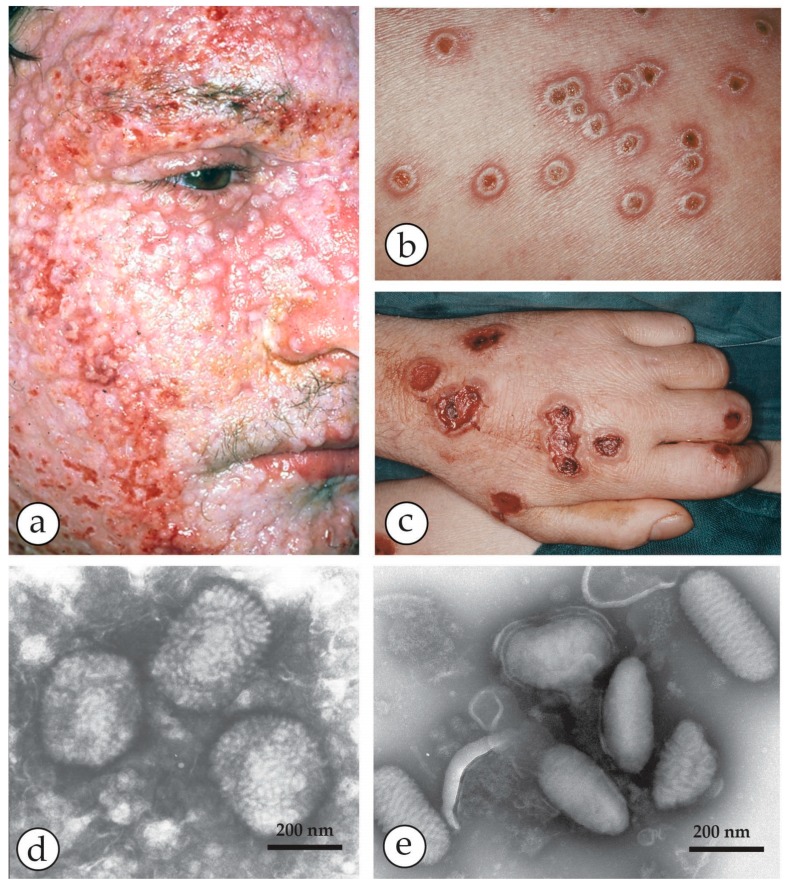
A fatal case of a cowpox zoonosis: (**a**–**c**) A zoonotic CPXV infection in an 18 year old immuno-compromised man. He was under massive steroid therapy for an allergy. The patient took care of a stray cat and developed fever and a generalized rash 10 days later. Vesicles developed into hemorrhagic pustules (**b**) with a tendency to fuse into larger ulcers (**c**). The patient died with circulatory collapse. (**d**) NS-DEM of specimens with PTA revealed brick-shaped particles with short surface threats typical of OPV. (**a**–**d**) reprinted from [33], with kind permission of Drs. Anna M. Eis-Hübinger and Bernhard Pfeiff and Springer Nature. (**e**) For comparison Parapoxviruses (PPV) after NS: smaller than OPV, ovoid and surrounded by long parallel spiral surface threads.

**Figure 4:**
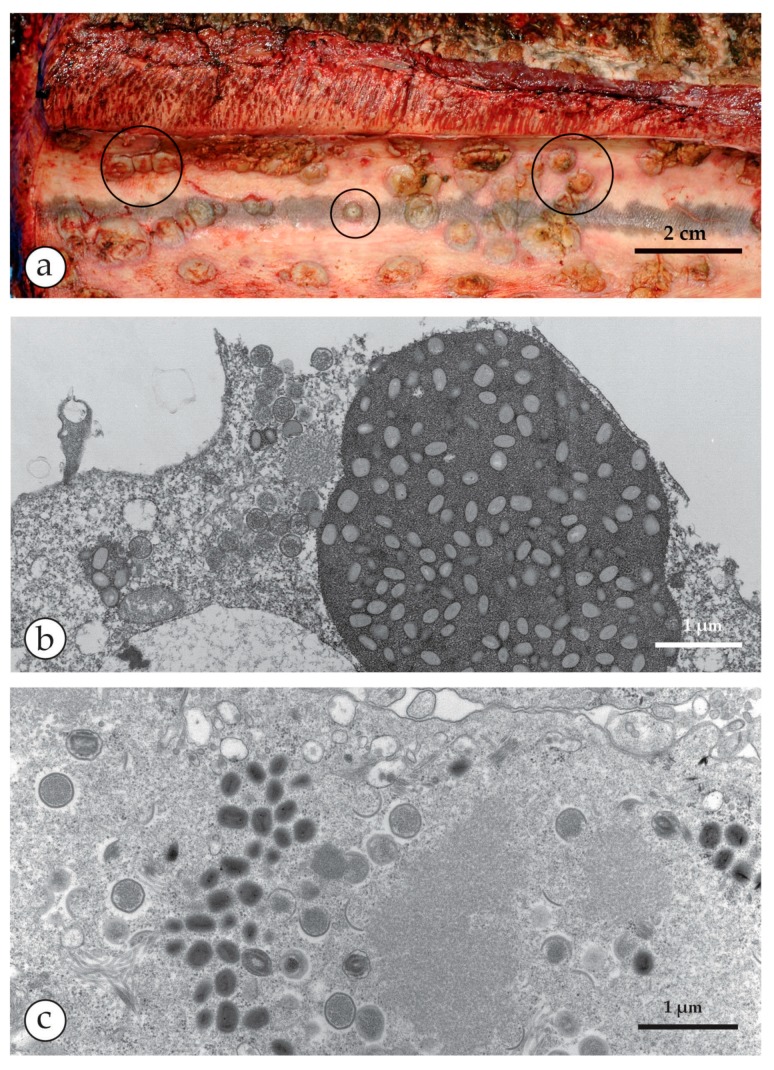
Peculiarities of CPXV infections: wide host range and specific inclusion bodies. (**a**) Lesions on the inner surface of a trunk of a deceased elephant, sliced longitudinally showing multiple ulcerous lesions on the mucous membranes [34]. Image courtesy Dr. Gudrun Wibbelt, Berlin. (**b**) After inoculation from the elephant´s ulcers in diagnostic cell cultures, well-circumscribed inclusion bodies developed consisting of a moderately dense matrix that included numerous mature virions. These are type A eosinophilic inclusions as seen in light microscopical histology and called Downie or Marchal bodies. They are typical of CPXV but found with a few other poxvirus genera [35]. (**c**) OPV are assembled in large cytoplasmic “factories” but these are basophilic (type B inclusions or Guarnieri bodies) in light microcopy and less well circumscribed.

**Figure 5:**
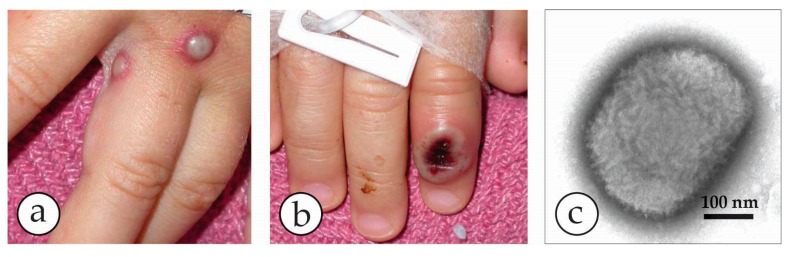
Human monkeypox in the USA in 2003 [36,37]. (**a**) The 2003 multi-state outbreak of human monkeypox in the Mid-West of the USA was initiated by an imported Gambian giant rat carrying MPXV. The rat infected prairie dogs kept as pets and the virus was transmitted to humans as zoonotic infections. The patients developed mild fever, lymphadenopathy and localized lesions where their pet animals had bitten or scratched them. (**b**) Vesicles developed into deep-seated pustules and slightly hemorrhagic ulcers that dried later. (**c**) The etiology remained unclear for 10 days until DEM was used, revealing typical OPV particles. Based on this orientation the final diagnosis of MPXV was confirmed at CDC [37]. Reprinted with kind permission of New England Journal Medicine.

**Figure 6:**
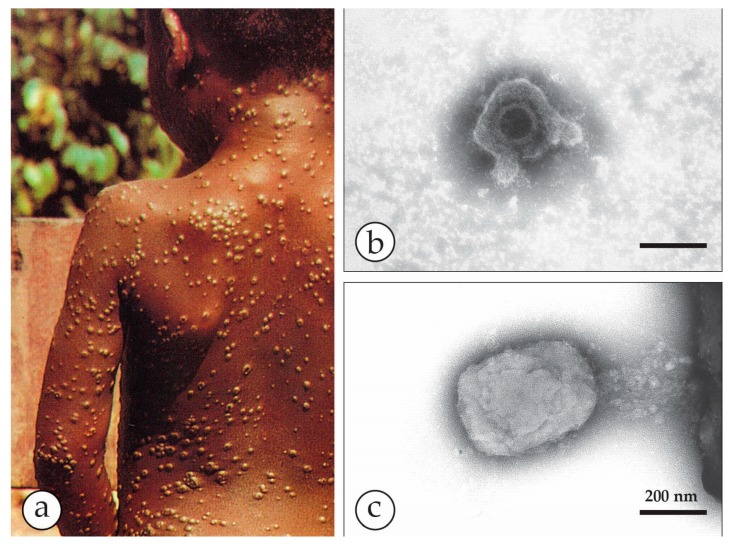
Outbreaks of human MPXV infections in Africa: (**a**) A boy from Democratic Republic of Congo with a generalized MPXV zoonosis. The vesicular lesions appear solid and are nearly all at the same developmental state (Photo courtesy of Mark Szczeniowski, WHO). After smallpox had been eradicated, human MPXV raised major concerns as an emerging zoonosis. Thirty years after the end of smallpox vaccination, the rate of this zoonosis increased 20-fold, in part due to increased contact with infected bushmeat [25]. Laboratory diagnosis showed, however, MPXV being responsible for only half the suspected “zoonoses”, the other half being caused by VZV [38,39]. DEM run in parallel at the Bernhard-Nocht-Institute in Hamburg by Christel Schmetz and at the Robert Koch Institute in Berlin confirmed the diagnosis. The samples for DEM had been kindly given by Prof. Hermann Meyer, Munich as aldehyde-inactivated samples and less than 5 µL each. Nevertheless, the particles shown in (**b**,**c**) have clearly typical herpesvirus and OPV-morphology respectively. Bars = 200 nm.

The OPV include—besides the *variola virus* (VARV) itself, which is now extinct in the field—*vaccinia virus* (VACV), the virus originally used by Jenner late in the 18th century to vaccinate against smallpox and which may have evolved from *cowpox* and *horsepox viruses* [1,15,16]. The widely used VACV “escaped into the wild” and variants of VACV are now globally endemic and are known as *buffalopox virus* (BPXV) [17,18,19,20,21,22,23]. As well as these, there are poxviruses native to animal species, some of which may infect man but without causing an epidemic. These less pathogenic OPV, as well as VACV, include *cowpox virus* (CPXV) [24], *monkeypox virus* (MPXV) [25] and *camelpox virus* (CMLV) [26,27]. All of them exist in distinct clades and can cause small, regional zoonotic outbreaks even with secondary and tertiary transmissions [23,28,29]. Other members of OPV such as *ectromelia virus* (mousepox), *squirrel-* and *volepox virus* have not been shown to cause infections in humans. In immunocompromised patients, however, all OPV (and other febrile rash agents) can cause severe generalized rashes and systemic disease. The clinical appearances in man and the associated viruses as seen in DEM are shown in Figure 1, Figure 2, Figure 3, Figure 4, Figure 5 and Figure 6. OPV virions are large by virus standards and are brick-shaped with short surface protrusions.

**Figure 7:**
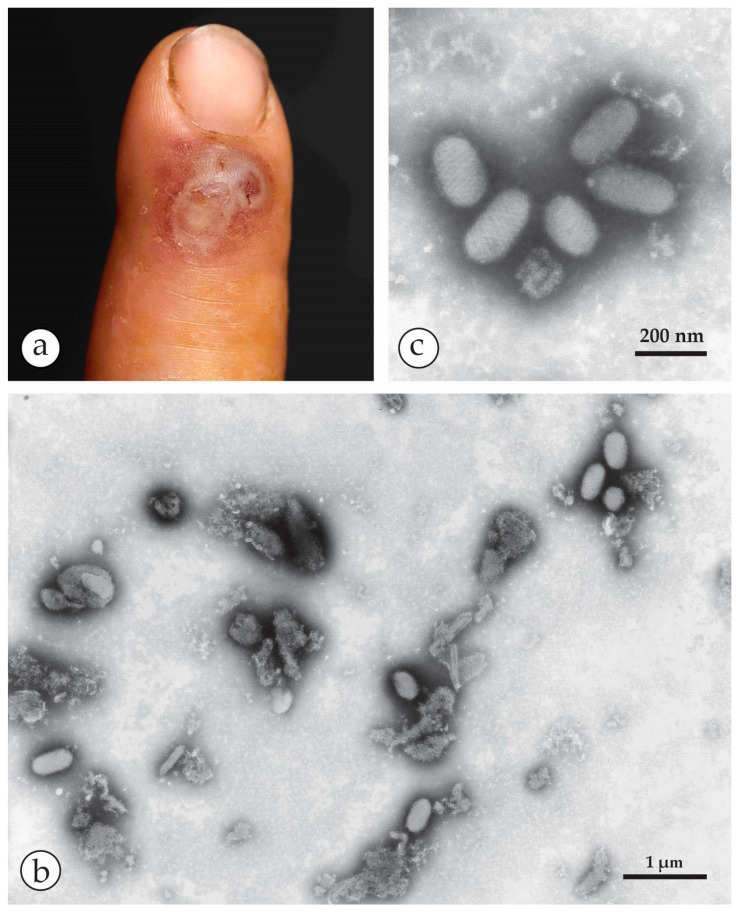
DEM of an Orf-zoonosis: (**a**) Index finger with a confluent haemorrhagic ulcer of a farmer who had handled his Orf-infected sheep [40]. (**b**) The “roof” of a lesion was homogenized. After NS with PTA ovoid particles are readily detected among some cellular detritus. The number of particles observed is consistent with a concentration of 10^7–8^ particles per mL in the original specimen. (**c**) Same case: The five virions shown at higher magnification are typical members of the PPV genus: they are smaller than OPV or MCV, differ by having an ovoid shape and present long, parallel running apparently spiral surface threads, Sample and clinical image courtesy of Prof. Klaus Eisendle, Bolzano). (**b**,**c**) reproduced from [40] with kind permission of Elsevier.

**Figure 8:**
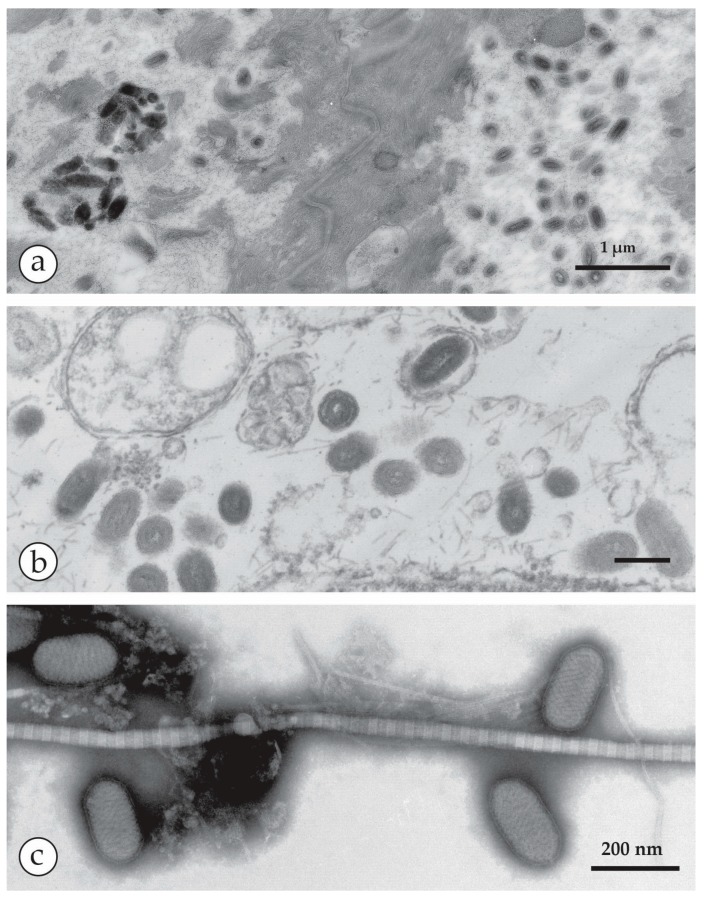
DEM of another human PPV infection (sample courtesy of Prof. Friedrich A. Bahmer, Bremen): (**a**) TS-DEM of a biopsy of a papule showing numerous particulate objects: on the right, PPV particles can be seen while the numerous electron-dense structures on the left are melanosomes–normal skin constituents Bar in (**a**) = 1 μm. In the middle are desmosomes connecting cells in the prickle cell layer. (**b**) TS-DEM at an intermediate magnification reveals the oval shapes typical of PPV seen in a damaged cell. (**c**) PPV particles are seen also by NS-DEM after grinding parts of the biopsy in distilled water, followed by low speed clarifying centrifugation. Amongst some detritus and a long collagen fibre, the ovoid PPV particles show long parallel surface threads. Bars in (**b**,**c**) = 200 nm.

The *Parapoxviruses* (PPV) are animal viruses that may be transmitted to man, causing a mild febrile zoonosis and single, nodular lesions containing some vesicular fluid. In immunocompromised patients they, too, may generalise. PPV include the species *Orf virus* (in sheep and goats), *Pseudocowpox virus* (in cattle, Milker´s nodule virus) and *Bovine papular stomatitis virus*, also in cattle. The clinical appearance in man and the associated viruses are shown in Figure 7 and Figure 8. The virions are slightly smaller than OPV, have a more oval outline and long, spiral surface “threads”.

**Figure 9:**
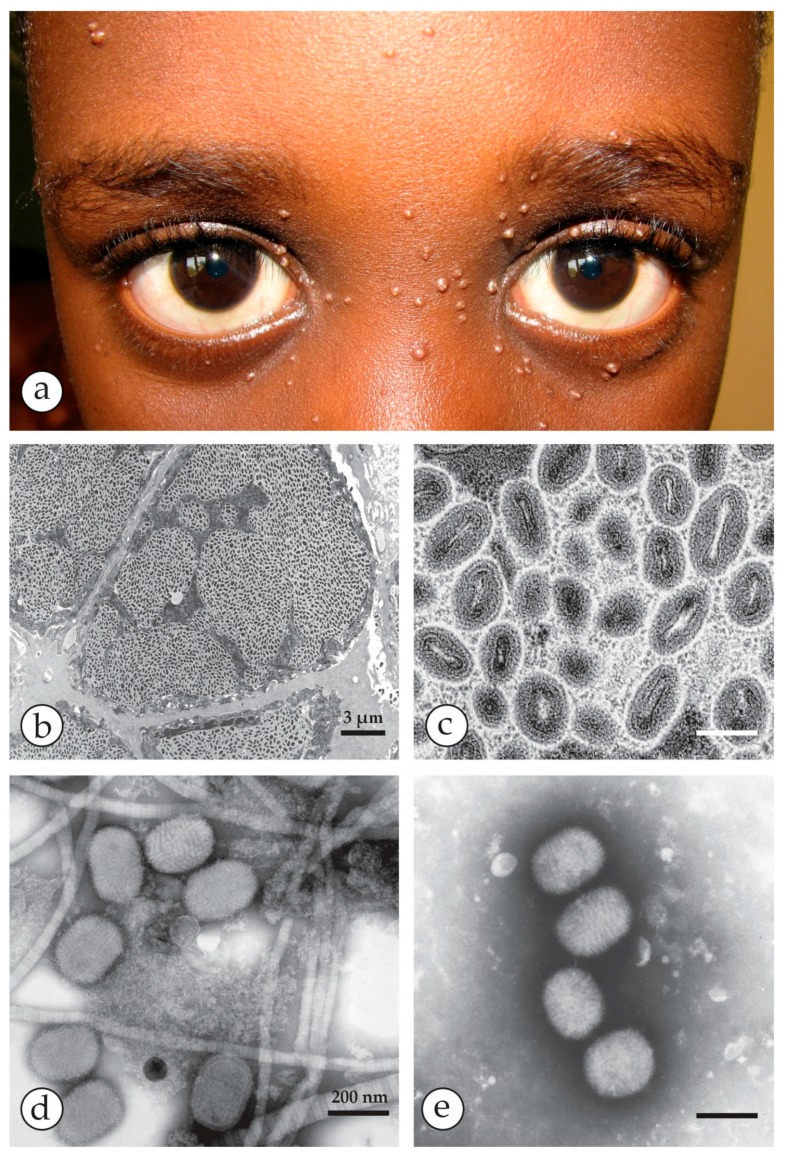
Molluscipox virus lesions: (**a**) Face of a young boy from Tanzania presenting numerous solid, yellow-whitish skin nodules typical of Molluscum contagiosum (photo courtesy of Prof. Constantin Orfanos, Berlin). (**b**,**c**): Ultrathin section TEM (TS-TEM) of a biopsy of a MCV papule. While the low power micrograph (**b**) shows the abundance of virus particles in the lobulated compartments of the nodule, the higher magnification in (**c**) reveals different orientations of MCV virions. By TS- and by NS-DEM, MCV are indistinguishable from OPV. (**d**) and (**e**): NS-DEM of the contents of MC papules: (**d**) After grinding a biopsy, six virions are shown which by size and shape and by the irregular surface structure closely resemble OPV. The virions are seen amid a meshwork of cell remnants and collagen fibres, the latter with their typical repeat pattern of 67 nm. (**e**) DEM without taking a surgical biopsy: the waxy content of a MC nodule was extruded by gentle squeezing using forceps. After dilution in distilled water, numerous brick-shaped particles, free from cellular contaminants were seen. DEM on many other skin lesions can be performed without using surgery [44]. Bars in (**c**–**e**) = 200 nm.

Two other poxvirus genera may be involved in human disease: (1) the widespread human-specific *Molluscipoxvirus* (MCV) causing single or multiple small wart-like lesions in man, called molluscum contagiosum (Figure 9); and (2) *Yatapox* virus, which is an occasional cause of a single skin lesion and is found in the tropics [41]. MCV infects man alone and is spread through direct contact or through contaminated clothing, towels, etc. By routine DEM, both viruses are indistinguishable from OPV. New animal poxviruses are still being discovered, with or without the potential for causing a zoonosis [42,43].

**Figure 10:**
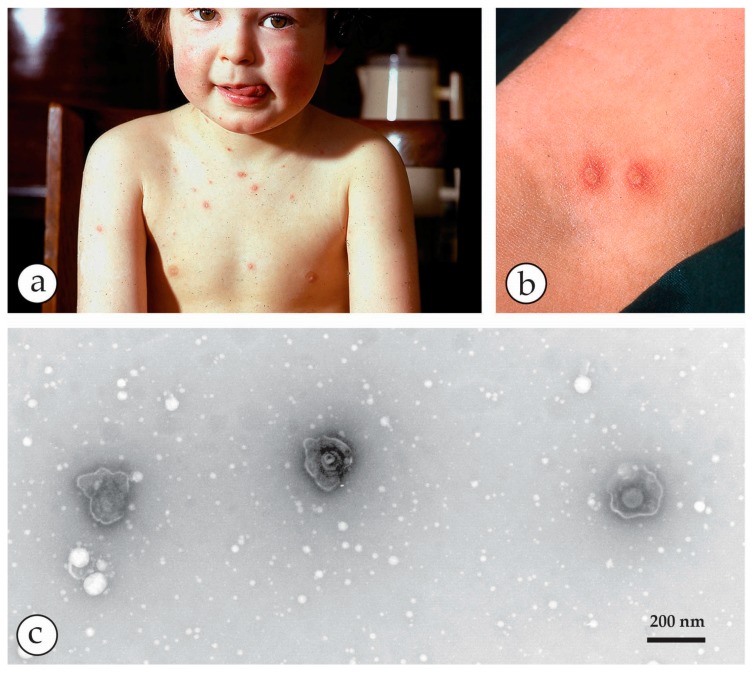
Chickenpox, a common, mild febrile rash in childhood. (**a**) Clinical chickenpox in a five-year old child. The lesions are mostly on the trunk. (**b**) Close-up of vesicles on the arm. They are more superficial compared with the deep-seated OPV or specifically those of smallpox. (**c**) NS-DEM of the clean vesicle fluid shows three herpesviruses. The labile virions are penetrated by the UAc stain and reveal ruptured envelopes containing the hexagonal central 110 nm capsids.

**Figure 11:**
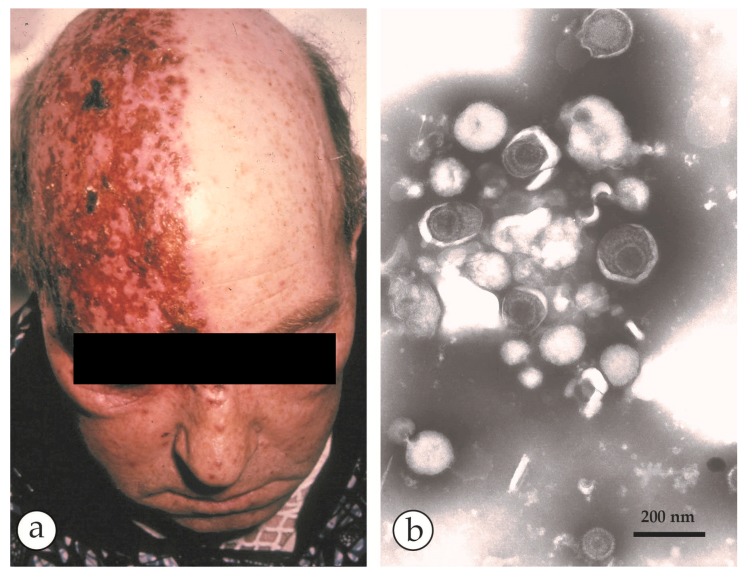
Shingles: (**a**) A case of shingles, a reactivated VZV infection, with its typical unilateral distribution of the vesicles, usually confined to a single dermatome. (**b**) Fluid collected from a vesicle later in the illness shows an aggregate of herpesvirus particles, penetrated by the PTA-stain, and mixed with some cell detritus. Combining DEM results with the clinical appearance and distribution of the lesions confirms the diagnosis of a reactivated VZV infection.

**Figure 12:**
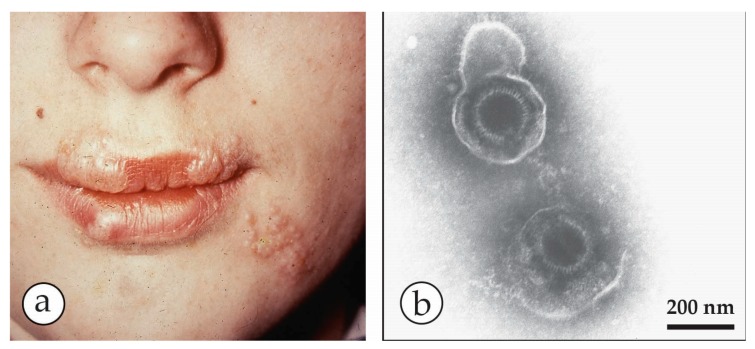
Herpes simplex eruption. (**a**) Severe herpes simplex eruptions on the lips and chin of a young woman. (**b**) Typical herpes virus particles seen after PTA NS of vesicle fluid. The labile envelopes are stain-penetrated and reveal the similarly stain-penetrated capsids. In the thinner stain, the upper virion also shows glycoprotein spikes in the periphery of its envelope.

**Figure 13:**
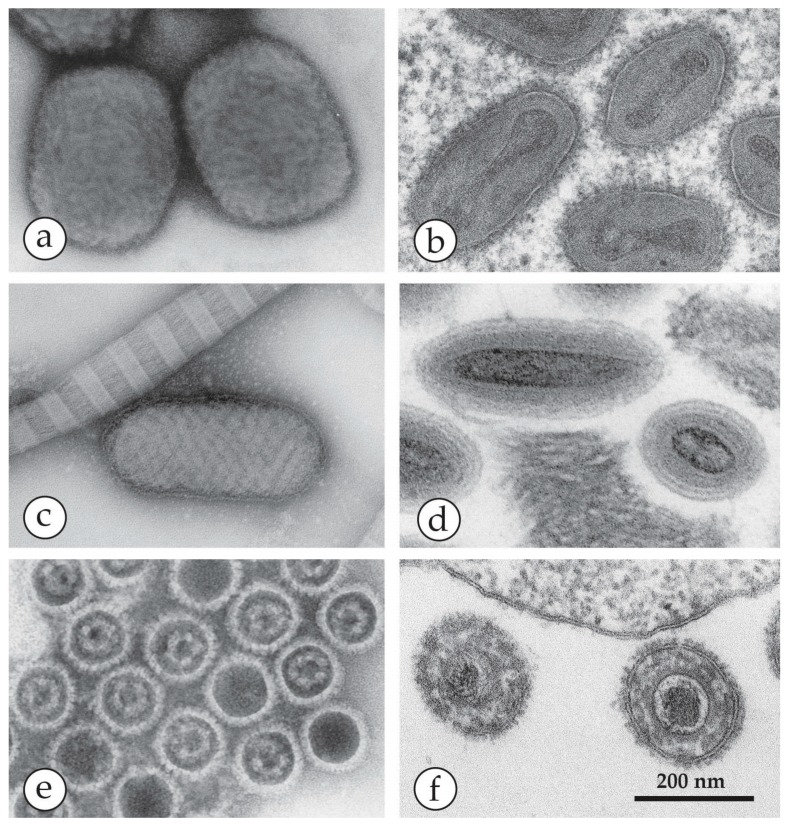
A comparison of OPV, PPV and herpesviruses after NS- (left) and TS- (right) preparation and DEM: (**a**) NS-DEM using UAc of a suspension of ectromelia OPV showing “brick-shaped” particles, 250 × 350 nm in size, with an irregular pattern of short, 10–15 nm surface protrusions. (**b**) TS-DEM of OPV from a diagnostic CAM from the last smallpox case in Germany [30]. Within the sections through the virions, inner components, the dumbbell-shaped core and lateral bodies can be seen. (**c**) NS-DEM of PPV observed in a diagnostic biopsy after grinding and NS with UAc. The ovoid virion is surrounded by parallel-running surface threads and lying beside a thick thread of collagen with its typical 67 nm-periodicity. The case was diagnosed later as a zoonotic infection of a butcher by bovine papular stomatitis virus. (**d**) TS-TEM of PPV observed in the biopsy from the same case as (**c**). In longitudinal section planes, PPV appear slimmer than OPV and they also lack the prominent lateral bodies of OPV. (**e**) NS of herpes virus capsids, showing occasionally a roughly hexagonal outline. The electron-translucent portions inside the core contain nucleoproteins associated with the viral DNA. (**f**) TS-TEM of a diagnostic culture showing two virions of equine herpesvirus (EHV-1). The envelope is studded with fuzzy surface projections. Underneath the lipid bilayer the ill-defined tegument and the slightly angular cores are seen. The latter contains the electron-dense viral genome.

**Figure 14:**
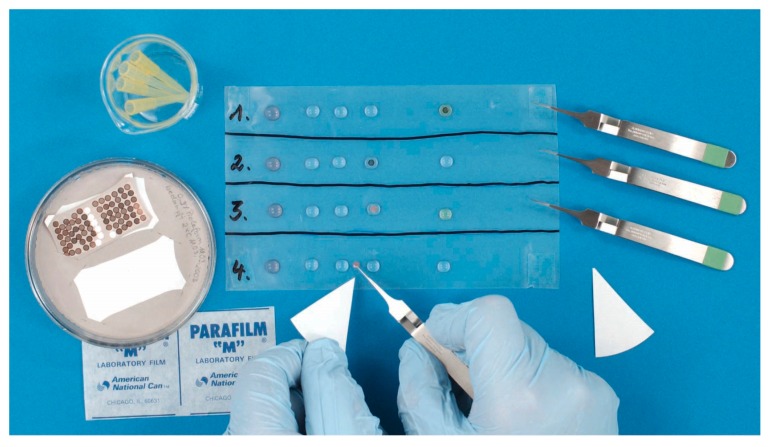
NS, the most rapid technique in diagnostic virology. It is done using a non-wetting surface, such as a sheet of Parafilm^TM^, and comprises the following steps: adsorption, washing and staining. A hydrophilic EM support grid is placed on a droplet of the diagnostic suspension (left) and after 30 s of adsorption is transferred quickly onto a series of droplets of distilled water to remove interfering salts, and then onto a droplet of “stain”. The “stains” used are electron-dense solutions: 0.5 to 2.0 percent of a heavy metal salt, such as PTA, routinely buffered to pH 7.2 (may be used between pH 5 to pH 10), UAc, unbuffered at a pH around 4.2, or other stains can be used. During a short, 5–10 s staining step, the stain does not react with the chemical moieties of the biologicals on the grid, i.e., there is no “positive staining” effect as there is in thin section-TEM. This rapid process results in “negative contrast”, as the transparent biological structures of the sample, after drying, are closely surrounded by the electron-dense stain. Reprinted from [45] with the kind permission of Springer Nature.

The Herpesviruses. Two virus species of the order *Herpesvirales—*varicella zoster virus (VZV) and herpes simplex virus (HSV) of the subfamily *Alphaherpesvirinae*—frequently cause infections in man. VZV is the cause of the common, and usually mild, chickenpox in children (Figure 10). During the primary infection, VZV enters the sensory root ganglia of the central nervous system where it remains dormant and may emerge again later as shingles (herpes zoster), as shown in Figure 11. This is usually limited to the distribution of one or two sensory nerves, as a painful vesicular eruption with, occasionally, a viral encephalitis. In contrast, there are two types of HSV—Type 1 causes small numbers of vesicles, often on the lips, as Cold Sores (Figure 12), while type 2 is a genital infection. Generally, type 1 occurs above the waist and type 2 below (genital infection, sexually transmitted) but either may be found anywhere on the body. Both infections are normally benign, though irritating to the patient, and often recur. The virions of the two types are indistinguishable by EM. In the immunocompromised, however, both VZV and HSV may cause serious life-threatening infections (Figure 13). Herpesvirus particles contain an icosahedral capsid core, 110 nm in diameter, which contains the DNA genome. This capsid is readily identifiable by its size and shape (Figure 6b, Figure 10, Figure 11, Figure 12, Figure 13b,c and Figure 14e,f) and is surrounded by an amorphous protein coat called the “tegument” and a loosely fitting envelope, 150–180 nm in diameter. The electron-dense stain used in DEM preparation often penetrates the capsid, giving a dark, “empty” appearance. The clinical appearances of chickenpox, shingles and cold sores, with their associated viruses, are shown in Figure 10, Figure 11, Figure 12 and Figure 13.

The enteroviruses are small RNA-containing viruses that may occasionally cause small epidemics of “hand, foot and mouth” disease—small aphthous ulcers in the mouth and vesicular lesions on hands and feet. However, the amount of virus in the lesions has not been shown to reach EM-detectable levels.

Other organisms, and none (see also Table 1): Anthrax can cause cutaneous skin lesions, usually a single “ulcer”, which evolves into a black scab later. It is not likely to be confused with smallpox, except in an immunocompromised patient. The lesions contain numerous large gram-positive rods, often containing a central spore. The treponemes of syphilis may also cause a single red papule, which later ulcerates. Similarly, allergic and drug-induced reactions may present as vesicular eruptions, as also may scabies and generalised dermatitis. All these may be substantially worse if the patient is immunocompromised.

## 3. Smallpox (Variola)—The Disease

Around 12 days after contact, the disease of smallpox presents as a fever, which relents as the vesicular rash appears after two to three days. The vesicles are more deep-seated than those of the herpesviruses, with thicker walls (Figure 1c), but, as listed in Table 1, other conditions—both infective and non-infective—may resemble smallpox to a greater or lesser extent. Given the widespread anxiety that would follow the reappearance of a variola-like disease, it would be necessary for health authorities to investigate and confirm the cause of any suspicious events [5,11,12]. To illustrate the danger, a general practitioner (family doctor) in Burnley, England in 1959 happened to mention to Allan Downie, a poxvirus expert, that, “the chickenpox seemed more severe this year”. Downie decided to investigate and found that the cause was not VZV at all but alastrim, the minor form of smallpox (A.W. Downie, pers comm). This episode did not then cause the alarm that it would now in the twenty-first century, 30 years after the cessation of routine vaccination.

Smallpox mortality varies between 30 and 80 percent [1]. If a smallpox-like disease occurred, there would be a need to establish a safe and certain diagnosis without delay—either it is variola (or a close relative), or another skin infection unlikely to cause an epidemic. The chance of variola virus being the cause may now be very small but is not non-existent [5,11]. A rapid diagnosis will be required and virologists must be able to provide it. This paper explains why electron microscopy should remain an indispensable constituent of diagnostic virology.

## 4. Diagnosis of Vesicular Skin Rashes

In the vesicle fluids contained in rashes caused by OPV or herpesviruses, there are >10^7^ physical particles of the causative agent per mL. These levels easily exceed the limit of detection and efforts to identify the cause will inevitably focus on this material [46,47,48,49].

Although Helmut Ruska had previously demonstrated clear-cut morphological differences between OPV and herpesviruses in Germany in 1943 [50], DEM was only used for the first time in 1948, in the New York smallpox outbreak by Nagler & Rake and independently by van Rooyen & Scott, who studied samples from India sent to Canada [51,52]. With the development of more advanced and easy to use electron microscopes and the negative contrast technique by Brenner & Horne in 1959, DEM was subsequently applied widely and successfully to smallpox diagnosis [1,48,53,54,55,56,57,58]. Although poxviruses were first visualized 80 years ago [59], the principle of DEM, the rapid visualization of known and unknown agents using simple and rapid preparation techniques, is still valid. Automated pattern recognition techniques are being developed and, supported by telemicroscopy, they could help to spread the global availability of DEM [60,61,62,63]. The relative lack of sensitivity by DEM can be partially corrected by using immune EM; broadly reactive hyper-immune sera as well as monospecific antibodies are available and can be used to advantage [64,65,66].

From the 1960s to 1990s, NS-DEM flourished when it was applied not only to examining the relatively clean fluids from suspect skin lesions but also to more “dirty” specimens such as faeces, urine, extracts of diagnostic cell cultures and solid-tissue infections containing more irrelevant debris [46,67,68,69,70,71,72,73,74,75,76,77,78,79,80,81,82,83]. In the twenty-first century, the further development of immunoassays (such as ELISAs), molecular methods for nucleic amplification techniques (NAT, e.g., PCR, sequencing and next generation sequencing) have shifted the focus of diagnostic virology to identifying a viral cause to the specific genotype direct from the specimen but only if the appropriate primers are available.

### 4.1. Unique Advantages of DEM in Rapid Viral Diagnosis

The strengths of DEM are that: (1) no specific reagents, no antibodies, no nucleic acid primers, nor any a priori decisions on what microorganisms to look for or which test to use, are required; (2) only the neutral (i.e., unbiased) sense of vision is used to recognise specific structures by their appearance alone; (3) DEM covers all the known (and even the unknown) agents; (4) can detect when there is more than one virus in the specimen; and (5) and most importantly, it is quick. The practical limits of resolution of a transmission electron microscope (TEM) on biological material is 2 nm, allowing the fine structures of any virus particles to be clearly visible. With this level of resolution, DEM is a catch-all method, able to detect unexpected viruses and other agents including bacteria and some parasites. DEM offers both speed and diagnostic certainty [46,64,68,69,70,73,75,76,84,85,86], while also allowing some of the other possible causes to be confidently discarded.

Speed in diagnostic virology matters in many instances—in severe, life-threatening clinical infections, in possible epidemic situations, as well as in possible bioterrorism. Currently, point-of-care or bed-side detection assays are being developed for many infections to enable diagnosis in less than an hour [87,88], always provided they contain the specific reagents necessary for detecting the actual causative virus. However, in contrast to these point-of-care systems, dedicated high tech methods like DEM will only be found in a small number of sites, mostly in universities and in centralized national facilities for cost and organisational reasons, but the “open view” of DEM is a valuable defence against the unexpected and, occasionally, double infections. Moreover, certainty over the diagnosis follows when the DEM result and the clinical history are compatible.

With skin diseases, several different specimens can be investigated by NS-DEM: vesicle fluids directly from the patient´s skin lesions, supernatants or cells from diagnostic culture, or solid tissues such as scabs or biopsies. The latter two can be evaluated by NS-DEM after grinding in distilled water and clarification with low speed centrifugation. TS-TEM can also be used in DEM, but preparing thin sections is more complex and more time-consuming (for details see [65,74,89,90,91]), making the simple NS the preferable method for DEM. More sophisticated preparation techniques, such as the structure-preserving cryo-TEM, are not required to make the diagnosis and will diminish the essential speed advantage of DEM [73].

### 4.2. Specimen Collection and Preparation

#### Collecting Diagnostic Fluids from Vesicles

Vesicle fluid for DEM should be collected in parallel with samples for NAT, using sterile instruments and one of four different techniques (see below), after puncturing the surface using, e.g., a sharp injection needle. Vesicle fluid or material directly from the base or the “roof” of the lesion is collected, and it is prudent to collect at least three replicate samples: for DEM, for NAT, for cell culture or for any necessary confirmatory tests. Essential infection control rules must be observed during collection and transport. Collection with a swab or a sponge is quantitatively disappointing [47]. Alternative methods are:

1. Aspirate fluid from three lesions into a small tuberculin-type syringe with a fine needle (29 gauge is ideal). Carefully re-cap the syringe and place it into a transport container. Cave: Recapping syringes is strictly forbidden in the US for safety reasons (unless it is one-handed or done with a device to hold the cap).
2. Open the surface of a vesicle using a sterile (injection) needle. Collect fluid into a fine glass tube by capillary attraction and close the tube at both ends with dental wax. The wax is removed later in a Laminar Flow Safety Cabinet and the fluid is carefully expelled onto a sheet of Parafilm^TM^ using a small rubber bulb on the “clean” end of the capillary.
3. Open the vesicle as in 2 above. Press the middle of a sterile glass light microscopic slide onto the fluid, remove the slide, let it dry, and mark it on the reverse where the droplet has dried to make finding the sample easier. Place the slide in a Petri dish for transport. To protect the dried fluid during transport, two matchsticks are placed as spacers at the ends of the sample slide which is then covered with another plain slide. Both slides are then bound together using an elastic band at each end.
4. With an open ulcerated lesion or when the base of a vesicle is uncovered, touch a microscope grid briefly onto the base holding it with fine-pointed forceps. Place the grid with specimen side uppermost onto a filter-paper disc placed in a Petri dish. Cover before transporting to the laboratory.

Notes on these techniques:

The “direct touch technique” (example 4, above) appears particularly efficient because it is the prickle cell layer of the skin lesions that produces the virus. When biopsies are taken, the bottom and roof of the lesion will contain virus concentrations at least 10-fold higher than plain vesicle fluid [40,47]. However, this “direct touch” requires practice to avoid damaging the support grid, and should be used only by specially trained staff or experts in DEM.

**Figure 15:**
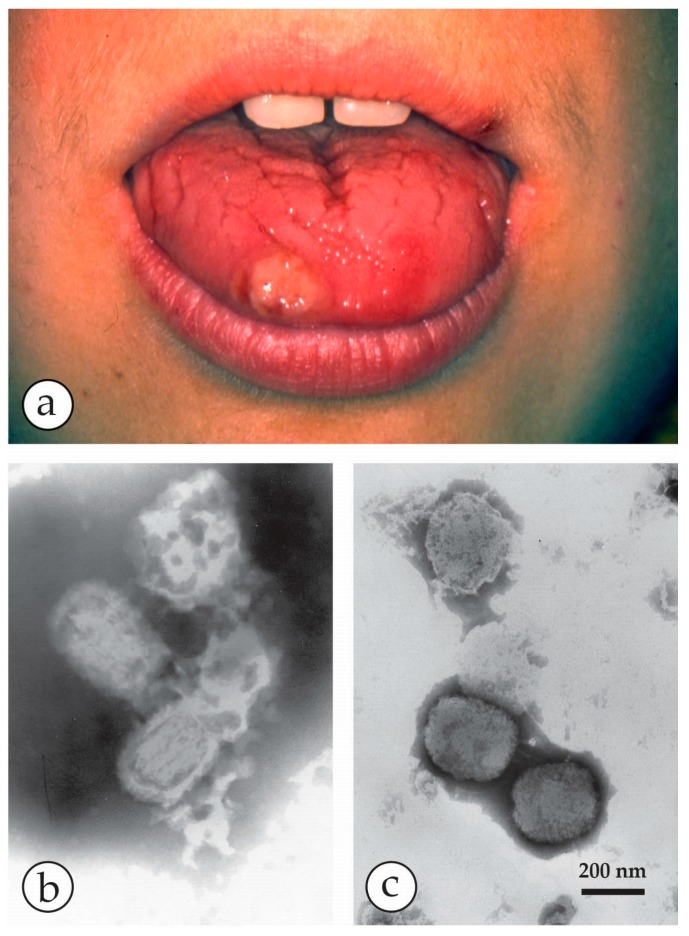
An unexpected result: (**a**) A presumed syphilitic lesion on the tip of the tongue of a young mother. When dark field light microscopy repeatedly failed to reveal *Treponema pallida*, doubts about the cause arose, and the patient was sent to hg for DEM to look for a viral cause. (**b**) Grids, touched onto the ulcerous surface, were stained with 2% PTA, pH 8.5, and subsequently inactivated by formaldehyde steam. Brick-shaped structures, 400 × 250 nm in size, were seen, most of them disintegrated and flattened. The internal “triple coil” (the DNA-containing inner body) is typical of OPV [92] (**c**) In the even stain, some surface detail and the overall size and shape are revealed. (**b**,**c**) are at the same magnification and reproduced from [93] with kind permission of Springer Nature.

The value of the “direct touch technique”, though, is shown in this example:

A young mother was sent to a Dermatology Poliklinik in Berlin in 1973 with provisional diagnosis of a syphilitic lesion on the tip of her tongue (Figure 15). Doubts were raised when no *Treponema pallidum* organisms could be demonstrated after several attempts and the patient was referred to hg. Direct touch specimens were taken from the surface (there was no vesicle fluid to collect) and the grids were stained with 2% PTA and disinfected at 60 °C with formaldehyde-steam for 30 minutes. An hour after the patient’s arrival, abundant brick-shaped structures, 400 × 250 nm in size, were seen in the EM. Most of them had disintegrated and become flattened (leading to an apparent increase in size), but still revealed details of an inner organization (Figure 15b,c). Despite the poor structural preservation, the size, shape and the internal “triple coil”, i.e., the DNA-containing inner body, are typical of OPV [92]. The explanation that emerged was that the patient’s daughter had been vaccinated recently and this was probably daughter-to-mother direct transmission [93]. The lesion was clearly not syphilitic and no anti-syphilis treatment was necessary.

The preferred technique to be used to collect such sample fluid should be agreed between the DEM lab and the respective hospital or Public Health institution beforehand and must be practised regularly in advance.

### 4.3. Specimen Support Grids for DEM

In contrast to high resolution work, grids covered with a robust support film and therefore stable in the electron beam, are required for DEM. Commercial 400 mesh copper grids with square holes, 30 × 30 µm in size, are most suitable. The grids should be coated in-house with a thin plastic film (preferably Pioloform^TM^ or Formvar^TM^), stabilized by evaporated carbon, and made highly adhesive (hydrophilic) for biological material by adding poly-lysine or alcian blue [94], or by glow discharge treatment [95]. These techniques and further procedures in DEM can be learned best during a visit to an expert lab or by attending a Special DEM Lab Course, such as the one offered by the Robert Koch Institute in Berlin [96,97]. Suitably prepared grids for DEM are also available commercially, though in-house production may guarantee better control of their suitability; it is important to maintain their hydrophilicity.

### 4.4. Negative Staining of a DEM Sample

NS—or “negative contrast”—is a method whereby an aqueous solution of a heavy metal salt is applied to a specimen. After air-drying on the grid, the stain forms a closely fitting, electron-dense “glass” around any objects on the grid. Biological materials are not electron dense and appear as lighter structures amidst the darker “stain”. Here, only the essential steps of the procedure will be outlined; more detailed information on its application in DEM is found in a number of papers and books [65,72,76,80,85,90,98,99,100,101,102]. Before preparation, the usual laboratory records will be initiated: the origin and the date/time of the diagnostic sample, as well as the patient’s ID, name, age and address, the clinical and, most importantly, the patient’s travel history and address (e-mail and phone number) of the sender.

In some cases, it will be necessary to release intracellular virus from a diagnostic cell culture by cycles of freeze-thawing, or by soaking crusts or biopsies in distilled water followed by grinding. Vesicle fluids and cell culture supernatants can be used directly for DEM. Low speed centrifugation (1000 *g* for 10 min) of cell-culture supernatants helps to achieve a more even staining by removing coarse debris. Using higher *g*-values is not recommended, as bigger structures, like OPV or virus-aggregates, are easily lost in the “high-speed” sediments. All procedures are performed under Biosafety Class 2 conditions. Samples should be inactivated, if required, and if possible even before entering the DEM laboratory or finally in the Biosafety Hood. Also, inactivated samples are processed further in the Laminar Flow Hood to protect the personal from any new, and possibly still active, germs (see under Biosafety, below).

A number of fine tipped forceps are required to handle the grids, while several micro-pipettes (Eppendorf or fine-drawn Pasteur) are used to handle droplets of the sample, double-distilled water for “washing” the grids, and the stain to be used. Small strips of torn-edged filter paper are used to draw off excess stain from the grid. The prepared grids are put either into special grid boxes (e.g., from LKB^TM^) or glass Petri dishes containing a filter-paper disc, for storing and transporting the prepared grids directly to the EM. It is vital that grids from different patients are clearly identified and kept separate, either in recorded positions in grid boxes, or in petri dishes laid out on filter paper labelled appropriately with a preparation number. Last but not least, means for safe disposal are required—a container with sodium hypochlorite to disinfect forceps and containers for safe disposal of fluid waste and solid materials must be at hand.

NS involves three steps—adsorption, washing and staining as shown in Figure 14**.** Droplets (30 µL) of the specimen are placed on a sheet of Parafilm^TM^, preferably in a humid chamber such as a large Petri dish containing a strip of wet filter paper. Instead of Parafilm^TM^, other non-wetting supports such as dental wax, clean glass microscope slides or even micro-titre plates may be used. Using forceps, a grid is laid onto the sample droplet. After a minimum of 30 s of adsorption, the grid is transferred to a succession of washing droplets (50 µL of distilled water) to remove salts reliably and some fine debris that can obscure the surface detail on any virus present.

For “staining”, solutions of 0.5 to 2.0 percent PTA at pH 7.2 (good contrast in the microscope) or aqueous UAc (lower pH, less contrast, but better fine detail) give the best results. The NS procedure consists of floating the grid with the adhering sample face down on the stain for a few seconds. Next, the grid is removed, surplus stain is drawn off with the torn edge of a piece of filter paper and placed face up on the filter paper in the Petri dish and covered. A few practical hints for preparation are:

1. To avoid cross-contamination use a different pair of forceps for each specimen. Always disinfect and clean the forceps carefully immediately afterwards.
2. The washing steps are helpful in getting an even distribution of stain on the grid. However, each washing step will appreciably reduce the number of particles on the grid.
3. Before staining with UAc, the grid with adherent specimen material must be washed on 3–5 droplets of distilled water to remove interfering phosphate ions. Successful NS with PTA does not require extensive washing; washing on a single droplet will suffice.
4. While checking the first grid in the TEM, the remaining specimen, the washing and stain droplets are left on the Parafilm^TM^, protected from dust and drying in the wet chamber. This helps to shorten preparation time in case further preparations, possibly stained differently, are needed.
5. Viruses and other biologicals will concentrate at the interface between the air and the sample fluid [103]. Letting the sample droplet remain untouched for a few minutes (or even longer, e.g., overnight at 4 °C in the refrigerator, if found necessary) before the grid is placed onto it for adsorption can help with low-concentration samples.
6. With sample volumes below 5 µL the sample may be placed directly onto the grid´s surface for adsorption.
7. While PTA staining tends to make biological structures more labile and porous, UAc is both a stain and a fixative [104]. When one stain does not result in a satisfactory preparation, the other usually does. Therefore, with unknown samples it may be advisable to use both stains in parallel. Preparations stained with either stain tend to deteriorate in the course of a fortnight. A loss of fine structure and the appearance of larger stain crystals (“grain”) can be avoided by storing “valuable” grids *in vacuo* in a desiccator containing some phospho-pentoxide as a desiccant. As well as these two common stains, a number of alternatives, e.g., ammonium molybdate, sodium silico-tungstate and uranyl formate have also been used successfully [90,98,105]. All three excel by a very fine “grain” and ammonium molybdate in addition by a well-balanced contrast.

It is good practice to check the magnification of the microscope. The instrument needs to be calibrated after every major service (e.g., on lenses) and a few representative magnifications should be tested. To confirm that the magnification is correct and to test the performance of the preparation method used, internal size and concentration markers, e.g., fixed catalase, other viruses, certified microbeads, or immuno-gold particles can be used [68,106].

### 4.5. Biological Safety in DEM

This aspect raises important questions. Preparative work for DEM is routinely done under Class 2 conditions, in a Class 2 Laminar Flow Cabinet, using protective clothing, gloves, goggles, etc. When unknown or highly transmissible agents may be involved, a risk analysis must be made following the detailed biosafety regulations laid down, e.g., by CDC, Office of Safety, Health, and Environment [107]. With cases of serious, life-threatening illness or from a post-mortem or where an agent liable to cause an epidemic may be involved, the samples should be inactivated using, e.g., a structure-preserving and efficient aldehyde disinfection scheme such as that detailed by Möller et al. [108]. The procedure takes 1 h (30 min at 25 °C/30 min at 37 °C) and damages neither viral fine structure nor virus detectability. When, however, the presence of smallpox or another life-threatening Class 3 agent is suspected, samples may have to be sent to a National Central Public Health Laboratory, where special safety precautions are in force to protect the public. The safe handling of organisms liable to cause a serious epidemic will be governed by local Governmental regulations, which should be observed, preferably with any course of action agreed beforehand.

## 5. Limitations of DEM

### 5.1. Cost and Availability of a Specific DEM Laboratory

DEM was largely discarded in routine viral diagnosis when highly sensitive and high through-put molecular diagnostic techniques were introduced. Today, DEM is used mainly to diagnose severe emerging infections and outbreaks of infectious diseases, to validate laboratory procedures and underwrite quality control (QC). The high costs of buying and maintaining a suitable EM and the salary of suitably qualified and dedicated staff are formidable barriers to continuing investment in DEM.

Given the advantages of DEM, current recommendations are still to make both NAT and DEM available as diagnostic techniques in parallel, in the event of a possible OPV outbreak [101,102]. This requires a positive decision to maintain DEM, supported by trained staff and in regular practice. Nevertheless, any decision to concentrate DEM to a single centralized facility has to be balanced against the time taken to transport specimens to this central facility when answers are needed very urgently.

DEM help can be provided if the responsible Local Public Health Institutions and Infectious Diseases Departments “recruit” suitable colleagues from nearby Institutes of Pathology or Biology and encourage them both to learn proper DEM competence and to develop good motivation. The missing expertise in DEM can be acquired by visiting a DEM expert laboratory as already mentioned. Today, emergencies demanding DEM are uncommon and a full-time commitment may not be necessary; it can be part-time and can be run in parallel with other activities, e.g., academic structural research and education or with other types of routine DEM, e.g., Good Laboratory Practice (GLP) control of production processes. Likewise, Centralized Imaging Facilities at Universities can also take on DEM when, in an emergency, a rapid catch-all diagnosis is required. In addition, DEM will help to shorten cell culture investigations in novel situations, as happened in 2003 during the SARS outbreak. DEM must be part of frontline defences and success is guaranteed only with good collaboration, such as in the Laboratory Response Network in the US and quality control measures in Germany [97,109,110].

### 5.2. Too Few Particles in the Specimen

Direct NS-DEM is possible when the initial particle concentration in the sample exceeds 1 × 10^5^ particles per mL [46,49,76]. In pox- and herpesvirus lesions, this value is exceeded, often by several orders of magnitude. With other samples, if there are too few particles on the microscope grid (i.e., fewer than one virus particle in a single 30 × 30 micron “window” in the support grid), it is possible to enrich the numbers. Direct sedimentation of particles onto the grid in an Airfuge^TM^, or equivalent, for 20 min increases the number of particles visible on the grid 50- to 100-fold [49,68,111,112]. Using specific antisera and direct sedimentation, suspensions with less than 1 × 10 ^3^ particles per mL in the original material have been positive by DEM [66]. A virus-specific antibody, if available, when bound to the grid, can enrich the number of detectable viruses, again by several orders of magnitude [66,113].

### 5.3. Lack of Dedicated and Experienced Staff for DEM

Firstly, DEM requires basic expertise in NS-preparation and how to operate the microscope. Lack of virological knowledge and pattern recognition ability can be rectified during visits to a qualified laboratory, by attending specific courses and with regular participation in an External Quality Assurance Program in DEM [97]. Stamina and persistence are required, too, because using DEM, in contrast to NAT and other internally controlled molecular diagnostic methods, is a skill. DEM cannot be run automatically on a machine; it means searching proactively for suspicious structures whilst not giving up too soon. DEM requires continuing interest and good pattern recognition abilities. By no means everybody who tries turns out to be a born “virus hunter”.

### 5.4. Low Sample Through-Put by DEM

While NAT, serological and other machine-based methods can run several samples simultaneously, DEM requires undivided attention for each individual specimen, although its “open view” does combine tests for all micro-organisms, viruses, bacteria and parasites, simultaneously [70,73,76,82,85]. With high particle concentrations on the grid, DEM works by immediate pattern recognition as a *prima vista* diagnosis (for example, “Numerous particles of OPV morphology present”) but still DEM is not a high through-put method. Lower concentrations of an inconspicuous agent will require enrichment techniques. Otherwise, a specimen can only be labelled “No virus seen” after a minimum of 20 min intense searching of several squares of the grid. Nonetheless, each examination is a comprehensive search for all possible causes at the same time. Preparing and examining more than 20 samples per day is a feasible work-load.

## 6. Conclusions

DEM has properties that make it invaluable in responding to a sudden infective threat, either accidental or deliberate. Its quick response, open view, certainty, and not requiring special reagents in situations out of the ordinary, show its usefulness when clear answers are needed urgently. For the diagnosis of febrile vesicular skin diseases, be they zoonotic OPV diseases or clinical emergencies in immunocompromised patients, DEM is particularly well suited as the diagnostic materials are easily accessible, without the need for taking biopsies [44]. In these situations, it is an essential component of the diagnostic service.

It is also important not to forget its unique potential to defuse fraught situations by showing that worst fears have not been realized. By being able to exclude the worst possible cause at an early stage, by finding something less dire, DEM has a particular value. Moreover, in an emergency, any good EM will do, provided the experienced staff and the instrument are immediately available.

**Figure 16:**
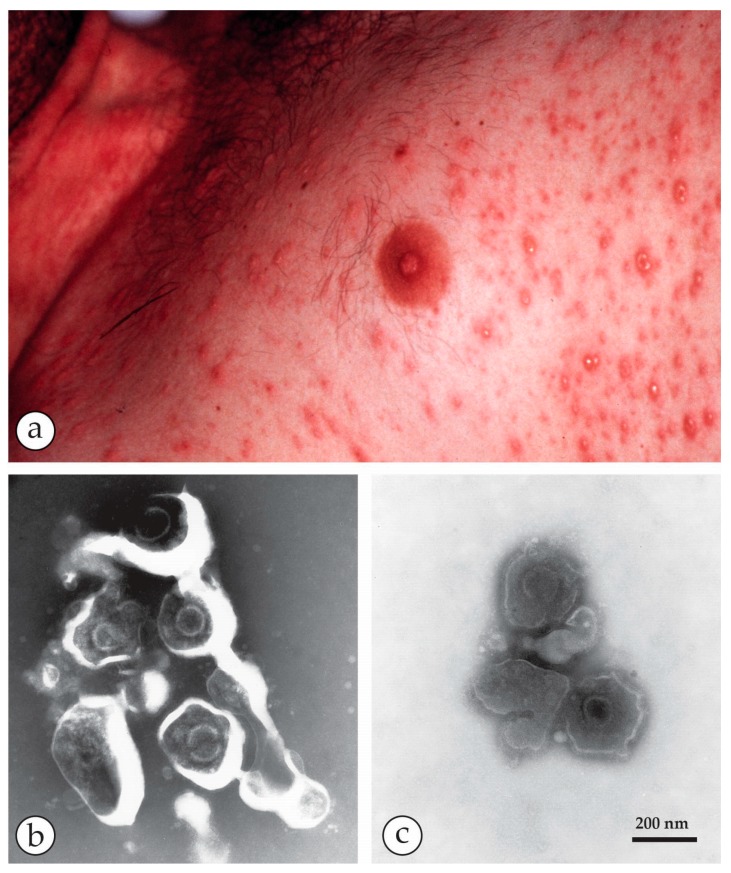
“An unconscious, unknown patient”: (**a**) Thin-walled blisters, differing in size and development on the thorax of an unconscious febrile patient. To collect fluid for rapid DEM, vesicles were opened and EM grids touched onto the vesicle´s base. (**b**) NS-DEM with 2% PTA revealed an abundance of herpesviruses. In the relatively ‘deep’ PTA-stain, the virions are morphologically degraded, i.e., the viral envelopes are broken open and some cores damaged by osmotic damage and shrinkage of the stain as it dries. (**c**) Three herpesvirus particles seen in a flat and even PTA-stain. Viral envelopes and cores retain the PTA stain as in (**b**). The measured diameter of the cores and the appearance of the envelope remnants allow a safe diagnosis: ‘particles of the order *Herpesvirales*.’ (**b**,**c**) are at the same magnification and reproduced from [73] (with kind permission of New Microbiologica).

DEM should be used as a front-line test in any case of suspected bioterrorism and to search for a (possibly) highly dangerous aetiological agent in severely ill and still undiagnosed patients. DEM may also be life-saving when normally less pathogenic agents are involved, as the following example shows:

On a late Friday afternoon in 1978, hg was called to see a patient in the Isolation Ward at the Virchow-Klinikum (opposite the Robert Koch Institute, RKI) in Berlin. He found a febrile unconscious middle-aged male patient with a vesicular rash and signs of raised intracranial pressure (Figure 16), and collected vesicle fluid at the bed-side directly onto microscope grids. Fifteen minutes later, NS-DEM at the RKI revealed numerous virus particles typical of the *Herpesvirales* order (Figure 16), showing that this was a case of adult chickenpox with an encephalitis—a severe primary VZV infection in an adult may be severe enough to resemble smallpox. In this life-threatening situation, DEM had excluded a poxvirus infection, however unlikely it may have been, as well as the allergic shock, which had been the clinicians’ provisional diagnosis. DEM had rapidly identified the true cause; the patient was treated with high-dose acyclovir and recovered completely. The speed, open view, certainty and the lack of a need for specific reagents for DEM had produced a result quickly enough to save a life, while at the same time excluding other, even worse, causes.

Last, but not least, without a proper hint of where to go with the diagnostics, clinical or environmental samples may turn out difficult to diagnose by NAT, especially when inhibitory substances are present [114]. Moreover, seeing the culprit provides a strong memory hook because virus morphology is easily remembered together with other pieces of structure-function information.

## 7. What is the Future for DEM?

The use of morphology-based diagnosis (the era of DEM) as a routine technique declined when highly sensitive and specific, high throughput diagnostic techniques, like ELISAs and nucleic acid amplification techniques (NAT, PCR, sequencing, and next generation sequencing), were introduced. Today DEM is considered by some critics as an expensive and imprecise method, dispensable in deciding treatment for the patient or further assessments required before assigning the virus to its place in classification. 

Electron microscopes are still in use in many life science-laboratories from Biology to Pathology, and yet more are used in Materials Science, whose “owners” may be even more reluctant to let infectious materials into their environment. Nonetheless, in an emergency, there is no overwhelming reason why suitably disinfected and prepared material might not be brought in to be examined given common sense and goodwill if discussed and agreed beforehand. The advantages of DEM of speed, certainty and the exclusion of some causes by finding a lesser one, should not be discarded lightly. Bioterrorism in its widest sense (deliberate or accidental) may be unlikely for practical reasons but is not impossible. Nature, too, can be an unintentional “bio-terrorist” by creating new, emergent viruses capable of initiating an epidemic, as recent events with SARS, MERS, Ebola and Zika viruses have shown. The time taken to make a diagnosis in these situations will always be an important factor. Not all viruses cause skin lesions, but DEM can be used to demonstrate the appearance of any agent. If it looks like an OPV, it will be an OPV. If it looks like a herpesvirus, it will be a herpesvirus. Although much of this article concerns the need and techniques of DEM to meet the threat of an outbreak of vesicular rash diseases, this is not the whole story of EM in virology.

EM in virology generally remains important. Emergent or re-emerging viruses must be fully characterized to establish their place in virus classification. Figure 13, which shows high resolution micrographs of representative OPV, PPV, and herpesviruses, demonstrates the detailed structure of each and their aesthetic charm as assembled micro-organisms. Virus structures arise from the economical use of their genetic information to code for structural subunits used repetitively to assemble the whole virion into recognizable morphological families, and the members of each family share many other properties—DNA or RNA, single or double stranded, the presence or absence of group antigens, replication strategy and even some indication of host range. Seeing the virus’s morphology will tell us much of its nature; from virus structure, other functional aspects can be reliably deduced.

Establishing the place of a new virus in the virus hierarchy will require more than morphology, but knowing what it looks like begins to put it into context. To classify an isolate as a type, or as something new in a clade of other types, nucleic acid sequencing will be required. However, we know since the time of Helmut Ruska [50,115,116] that demonstrating the fine structure of an agent allows it to be assigned to a specific virus family, in some situations down to the specific subfamily [75], and in diagnostic virology, a morphological diagnosis can be sufficient for the public health officials to rule out a suspected dangerous outbreak, and for clinicians to decide, for example, on life-saving therapy, as shown in the case of the VZV-infection in an adult. DEM, with its speed and certainty, remains a potent weapon in our defences against epidemic viruses, old or new.

## Figures and Tables

**Table 1:** Skin Lesions of Man and their infective or Non-Infective Cause.

Micro-Organism, Agent or Condition	Disease	Lesion Appearance *
*Orthopoxviridae* (OPV) Variola major virus Variola minor = Alastrim Man is only known host, currently eradicated as a human disease	Smallpox Alastrim	Generalised vesicular rash with large deep-seated vesicles all at the same stage, dimpled at the centre developing into pustules and crusting over later. Centrifugal distribution, including soles and palms. May be modified by previous vaccination
Other OPV: Vaccinia virus, buffalopox, cowpox, monkeypox, camelpox	Various animal diseases, occasionally transmitted to man	Usually single vesicular or papular lesion, developing into ulcer, and crusting later Lesions larger, up to 1cm in diameter, may not be clearly vesicular
*Parapoxviridae* (PPV) of goat, sheep: orf; cattle: paravaccinia, pseudocowpox	Animal diseases transmitted to man as Orf, Pseudocowpox (Milker’s nodules)	Large (up to 1 cm) nodular with little vesicular fluid, developing into an ulcer, crusting later May not be clearly vesicular
Molluscipoxvirus (MCV) specific for man. There is also animal specific MCV	Molluscum contagiosum warty lesions–may be multiple, may be passed as a sexually transmitted disease. Auto-inoculation may spread the lesions	Solid, firm, wart-like tumours: dome-shaped or flat. Pearly or flesh-coloured nodules with a depression on the top. Not clearly vesicular but contain waxy sacs packed with virus particles
Herpes varicella zoster (VZV)	Chickenpox, usually in childhood or Shingles (herpes zoster = recrudescence of previous varicella)	Generalised or scanty vesicular rash, mostly on head and trunk becoming pustular and crusting later. Lesions smaller, frailer and less deep-seated than smallpox, can be easily ruptured. Differ in size and stage of development Shingles: similar lesions but confined to the distribution of one or more sensory nerves.
Herpes simplex virus HSV-1, HSV-2	“Cold sores”, usually limited to a few localised lesions, usually on the upper lip. Very occasionally a herpes encephalitis. Herpes simplex may be sexually transmitted	Limited recurrent vesicular lesions with prodromal tingling Lesions smaller and less deep-seated than smallpox, crusting later.
Enterovirus and other small spherical RNA-containing viruses	Hand-foot-and-mouth disease	Aphthous oral ulcers and small vesicular lesions 2–4 mm in diameter on the hands and feet, can also generalize.
Anthrax, *Bacillus anthracis*	Cutaneous Anthrax	Single or small number of large vesicles later, surrounding a dark central crust (“Malignant pustule”)
Treponema pallidum	Primary and secondary syphilis	Single red papule 0.5 to 2 cm in diameter developing into ulcer with an indurated margin and exudate
Drug-induced rashes	A variety of rashes: exanthematous pustulosis, Erythema multiforme	No specific micro-organisms present
Scabies and insect bites	Variety of single or multiple quasi-vesicular lesions	No specific micro-organisms present
Contact dermatitis	Symptomatic toxic-dermatitis	No specific micro-organisms present

* Lesions in patients who are immunodeficient or immunosuppressed may be more florid and may become generalized.

**Table 2:** The *Poxviridae*: Genera of the *Chordopoxvirinae* of Medical Interest [13].

Genus	Members, Species	Disease in Healthy Men	Natural Host	Appearance in DEM and Size
*Orthopoxvirus* (OPV)	Variola virus (VARV)	Smallpox Variola major Variola minor	Man only	Brick-shaped virions, 250–350 nm × 200 nm with an irregular array of 10–15 nm surface “protrusions” (threads).
	Vaccinia virus (VACV)	Vaccination = local, self-limiting lesion	endemic in cattle and buffaloes in India and Brazil	
	Cowpox virus (CPXV)	Scanty vesicular rash developing into ulcer	Rodents transmitting CPXV to cattle, cats and other mammals	
	Monkeypox virus (MPXV)	Vesicular rash, similar to smallpox	Squirrels, non-human primates	
	other OPV: camelpox, buffalopox	Single or multiple vesicular-pustular lesions developing into ulcer	Various *	
	mousepox (ectromelia) and several others, some unclassified	no known disease in man		
*Parapoxvirus* (PPV)	Orf (ecthyma contagiosum)	Single tender nodule developing into ulcer 10–15 mm in size	Sheep, goats	Oval virions: 250–300 nm × 150–180 nm with long, spiral surface threads
	Pseudocowpox, Bovine papular stomatitis	see Orf	Cattle	
*Molluscipoxvirus* (MCV)	Molluscum contagiosum virus (MCV)	Single or multiple papules developing into pink fleshy “warts”, often with umbilicated centre	Man only	Brick-shaped virion, short threads: Very similar to OPV
*Yatapoxvirus*	Tanapox	Single or multiple firm nodules	Non-human primates	Brick-shaped, very similar to OPV

* Poxviruses have been found in other vertebrate and invertebrate species.

## Abbreviations

BPXV	Buffalopox virus
CAM	Chorio-Allantoic Membrane of fertile hen’s egg, used for isolating pox and herpes viruses
CDC	Centers for Disease Control & Prevention, Atlanta, GA, USA
CMLV	Camelpox virus
CPXV	Cowpox virus
DEM	Diagnostic Electron Microscopy
DNA	DeoxyriboNucleic Acid
ELISA	Enzyme-Linked Immunosorbent Assay (often abbreviated to EIA)
EM	Electron Microscope or Electron Microscopy
GLP	Good Laboratory Practice
HSV	Herpes Simplex Virus
MC	Molluscum Contagiosum
MCV	Molluscipox Virus
MERS	Middle East Respiratory Syndrome
MPXV	Monkeypox virus
NAT	Nucleic Acid Amplification Techniques
NS	Negative Staining
NS-EM	Negative Staining Electron Microscopy
OPD	Out-Patients Department
OPV	OrthoPoxVirus
PCR	Polymerase Chain Reaction
PPV	ParaPoxVirus
PTA	Potassium phosphoTungstic Acid
SARS	Severe Acute Respiratory Syndrome
TEM	Transmission Electron Microscopy
TS	Thin Section
TS-EM	Thin-Section Electron microscopy
UAc	Uranyl Acetate
VACV	Vaccinia virus, used for prophylaxis against smallpox
VARV	Variola virus, cause of Smallpox
VZV	Varicella Zoster Virus
